# Attitudes and Intentions toward COVID-19 Vaccination among Health Professions Students and Faculty in Qatar 

**DOI:** 10.3390/vaccines9111275

**Published:** 2021-11-03

**Authors:** Amine Zaidi, Amal Elmasaad, Hend Alobaidli, Rana Sayed, Dana Al-Ali, Dana Al-Kuwari, Shaikha Al-Kubaisi, Yosra Mekki, Mohamed M. Emara, Suhad Daher-Nashif

**Affiliations:** 1 Basic Medical Sciences Department, College of Medicine, QU Health, Qatar University, Doha 2713, Qatar; az1708239@student.qu.edu.qa (A.Z.); ae1800819@student.qu.edu.qa (A.E.); ha1704865@student.qu.edu.qa (H.A.); rs1703961@student.qu.edu.qa (R.S.); da1802872@student.qu.edu.qa (D.A.-A.); da1701937@student.qu.edu.qa (D.A.-K.); sa1601106@student.qu.edu.qa (S.A.-K.); ym1707134@student.qu.edu.qa (Y.M.); memara@qu.edu.qa (M.M.E.); 2 Biomedical and Pharmaceutical Research Unit, QU Health, Qatar University, Doha 2713, Qatar; 3 Population Medicine Department, College of Medicine, QU Health, Qatar University, Doha 2713, Qatar

**Keywords:** COVID-19 vaccination, attitudes towards vaccine, vaccine hesitancy, sociodemographic factors, knowledge, health professions students and faculty, Middle East

## Abstract

A population’s desire to take the COVID-19 vaccine is an important predictor of a country’s future pandemic management. This cross-sectional study examines the impact of psychological and sociodemographic factors on attitudes toward and intentions to take the COVID-19 vaccine among students and faculty at four colleges of health professions and sciences at Qatar University. The data were collected through an online survey using Google Forms. The survey was distributed through various online platforms. Data analysis was conducted using Stata 16. Of the 364 participants, 9.89% expressed a high mistrust of vaccine safety, and 21.7% were uncertain about their levels of trust; 28% expressed strong worries about unforeseen side effects, whereas 54.95% expressed moderate worries. Furthermore, 7.69% expressed strong concerns and 39.84% showed moderate concerns about commercial profiteering. Approximately 13% of the participants expressed a strong preference towards natural immunity, whilst 45.33% appeared to believe that natural immunity might be better than a vaccine. Importantly, 68.13% of the participants intended to receive the COVID-19 vaccine once it became available, compared to 17.03% who were uncertain and 14.83% who were unwilling to be vaccinated. Our findings differ from the data on vaccine hesitancy among the general population of Qatar. We argue that this gap is due to scientific knowledge and domain of education. Furthermore, although knowledge and awareness may affect vaccine attitudes, mental health and sociodemographic factors play a role in shaping attitudes towards vaccines.

## 1. Introduction

COVID-19 is an infectious disease caused by a newly discovered coronavirus strain known as Severe Acute Respiratory Syndrome Coronavirus 2 (SARS-CoV-2) [1]. Social distancing and mask-wearing have been the main control measures that have helped prevent and control the spread of the SARS-CoV-2 virus, and these have proven to be efficient [2]. In addition to these measures, many other global efforts and preventive measures have been applied with the aim of decreasing the negative outcomes of the COVID-19 pandemic [3]. One of these measures is the massive global application of safe and effective vaccines against COVID-19. Vaccination has been proven to play a major role in reducing the number of cases and complications since the beginning of its application. Therefore, governments have supported the intensive efforts of the scientific community and the pharmaceutical industry in developing safe and effective vaccines for COVID-19. For the vaccination process to be effective, governments and societies must uncover and understand the factors that influence a population’s attitudes towards vaccinations in general, as well as their readiness to be vaccinated against COVID-19. 

Despite the importance of COVID-19 vaccination and the success of its application worldwide, hesitancy to take the vaccine is common among various populations and across cultures and education levels. Indeed, the WHO declared vaccine hesitancy as one of the top ten threats to global health in 2019 [4]. According to the Strategic Advisory Group of Experts on Immunization, vaccine hesitancy refers to “a delay in acceptance or refusal of vaccination despite the availability of vaccination services” [5] (p. 4163). Present-day endorsement of vaccine hesitancy is a known phenomenon, with roots that have accompanied vaccination since its scientific inception. The phenomenon of vaccine hesitancy has resulted in the revival of some infectious diseases that could have been eradicated through mass vaccination, such as measles, poliomyelitis, and pertussis [6]. Vaccine hesitancy exists worldwide, crossing cultures, regions, and continents [7]. Recent studies reveal that COVID-19 vaccine hesitancy involves sociocultural and sociodemographic factors. In a systematic review of COVID-19 vaccine hesitancy worldwide, Sallam reported the impact of psychological and sociodemographic factors on attitudes towards and intention to vaccinate against COVID-19 [8]. Sallam argued that most studies on this topic have focused on assessing the attitudes and acceptability of the COVID-19 vaccine and the underlying reasons behind vaccine uncertainty and unwillingness [8]. For example, Paul et al. reported in their recent study that distrustful and skeptical attitudes towards the vaccine were higher amongst individuals from ethnic minorities and those with lower levels of education, lower annual income, poor knowledge about COVID-19, and poor compliance with government COVID-19 guidelines [9]. They also reported that individuals from ethnic minority backgrounds are the most vulnerable to falling ill and dying of COVID-19 and are less willing to be vaccinated. Furthermore, vaccine hesitancy and vaccine refusal were significantly associated with low education levels and poor compliance with recommended vaccinations in the past [10]. 

In addition to education and ethnicity, other socioeconomic factors such as age were shown to play a role in vaccine hesitancy: young adults (ages 18–29) have more positive attitudes towards vaccines compared to older adults (ages 65+) [9]. In contrast, Freeman et al. stated that vaccine hesitancy is associated with younger age groups [11]. They also found that vaccine hesitancy is more common among women and those with lower incomes [11]. Several additional studies have reported the impact of gender on vaccine-related attitudes and intentions. It was found that women were more likely to express concerns about the unforeseen side effects of vaccines than men [9]. In contrast, Largent et al. stated that no attitude differences could be observed between genders regarding the COVID-19 vaccine [12]. Further studies have also shown that the concerns people have regarding the vaccine are related to unforeseen side effects and a general mistrust in the benefits and safety of the vaccine [13,14]. The contradictions between the conclusions of these various studies can be explained by the gaps in the sociodemographic and socioeconomic factors of populations. In our study, we will report on sociodemographic determinants of attitudes and intentions to vaccinate against COVID-19 among young adults and middle-aged adults from both genders, and report on how these factors may shape vaccine willingness and attitudes, despite strong scientific knowledge on the topic. 

This study focuses on faculty and students in the health professions. In the majority of the recent studies on the issue, this group was included as part of the general population without examining its unique characteristics. Focusing on this group is of importance because health professions faculty and students are among the key knowledge agents in any society who contribute to shaping the knowledge and the awareness of their close communities and the general population. By conducting this study, we provide additional information to the existing resources on how knowledge and awareness contribute to shaping attitudes towards COVID-19 vaccination, and how sociodemographic factors interact with the scientific knowledge to influence vaccine attitudes and intentions.

While most of the existing studies mentioned that attitudes may change over time due to the COVID-19 vaccination rate and its positive impact on individuals and societies, there are still substantial groups in each society that reject or are hesitant towards the vaccine. These attitudes are influenced by sociocultural and socioeconomic factors. 

Few studies have addressed this issue in the Arab region, and even fewer have addressed it among health students and faculty. A study from Saudi Arabia found that of 992 respondents, 642 showed an interest in accepting the COVID-19 vaccine if it was available [15]. The willingness to accept a future COVID-19 vaccine was relatively high among older age groups, married participants with postgraduate or higher education levels, non-Saudis, and those employed in the government sector [15]. Another study on the general population in Lebanon found that of the 579 participants, 21.4% were willing to receive the vaccine, whereas 40.9% refused and the remainder were hesitant [16]. This study also found more vaccine hesitancy among married individuals than single individuals, and more hesitancy among women than men. In Jordan, the intention to get the COVID-19 vaccine was low, with 34.9% of participants agreeing to be vaccinated, whereas 39.6% were not willing to be vaccinated and 25.5% were indecisive. The acceptance rates of the COVID-19 vaccine were higher among men (42.1%) and students at health schools (43.5%) [6]. In a cross-sectional study on hesitancy among Arab health care workers worldwide, Qunaibi et al. found a significant rate of vaccine hesitancy among 5708 Arabic-speaking health care workers residing in and outside of Arab countries [17]. They reported that the highest rates of hesitancy were among participants from the western regions of the Arab world (Egypt, Morocco, Tunisia, and Algeria), and the most cited reasons for hesitancy were concerns about side effects and distrust of the expedited vaccine production and healthcare policies. Factors associated with higher hesitancy included an age of 30–59, previous or current suspected or confirmed COVID-19 infection, female gender, and the choice not to regularly receive the influenza vaccine. In a previous study among the general population of Qatar, Khaled et al. reported that of the 8323 participants in a phone survey, 42.7% were accepting, 45.2% were hesitant, and 12.1% resistant [18]. They also found that female gender, Arab ethnicity, migrant status/type, and vaccine side-effects concerns were associated with hesitancy and resistance. 

Although attitudes towards the COVID-19 vaccine have been addressed in the Arab region, none of the previous studies addressed them among health professions students and faculty. This study is thus the first of its type in the Arab region. The study aims to examine attitudes towards the COVID-19 vaccine and willingness to take the vaccine among health professions students and faculty of different nationalities in Qatar. The study also aims to assess the impact of sociodemographic factors and the mental health of participants on their attitudes and intentions regarding the vaccine. Our results show that being a health professions student or faculty influences both attitudes towards COVID-19 vaccination and willingness to take the vaccine. Although knowledge has an impact on attitudes and intentions, sociodemographic factors and mental state influence these attitudes and intentions. We believe that examining the predictors and factors that may affect willingness to take the vaccine among health professions students and faculty will help in building awareness campaigns tailored to the population’s needs. 

## 2. Materials and Methods

### 2.1. Setting

A total of 364 health professions students and faculty of different nationalities at Qatar University participated in this study. Generally speaking, Qatar’s population includes 94 nationalities, incorporating various religious and ethnic identities. As a part of the Qatar Vision 2030 and in order to implement the National Health Strategy, which is tailored to meet the increases in health demand and quality, Qatar University has established the Health Cluster [19], which includes the College of Health Sciences, the College of Pharmacy, the College of Medicine, and the College of Dental Medicine. 

The first case of COVID-19 was reported in Qatar in late February 2020 [20]. Following this first case, the number of cases increased, and the government imposed a lockdown in mid-March 2020. It has since become obligatory to wear masks in public places and download *Ehteraz*—a contact tracing application. Such measures have been effective, and cases started to decrease from May 2020 until February 2021. During this period, Qatar introduced four phases for lifting the lockdown and successfully implemented them. Furthermore, in December 2020, the Qatari government announced free COVID-19 vaccinations for the entire population, starting with the groups at high risk of infection including healthcare providers. At the time of writing this paper, 63% of the population over the age of 12 are vaccinated [21].

### 2.2. Sample Design and Participants

**Figure 1:**
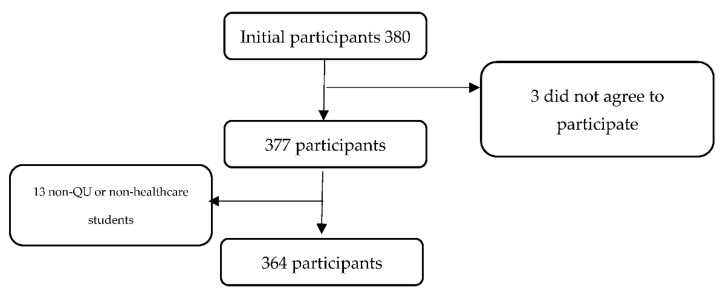
Flowchart for the screening of participants.

This cross-sectional study uses an online survey through the Google Forms platform. We included students and faculty from each of the health sciences and professions colleges that make up the Health Cluster at Qatar University. The inclusion criterion required participants to be currently enrolled or employed in one of the health colleges, including the College of Medicine, College of Dental Medicine, College of Pharmacy, and College of Health Sciences (CHS). CHS includes programs in Biomedical Sciences, Public Health, Human Nutrition, and the Physical Therapy/Rehabilitation Department. Biomedical Research Center researchers were also included. Our study was inclusive of both vaccinated and non-vaccinated individuals. The estimated total number of students in the QU Health Cluster was 1325, while the total number of faculty members and researchers (from the QU Health cluster and the BRC) were 208. The expected response rate was 50% and, therefore, we anticipated that 767 participants would answer the survey. The minumum number of subjects was calculated and set to be 65. The initial number of participants was 380. However, only 364 met the inclusion criteria and have completed the survey, giving an overall response rate of 23.72% (Figure 1) (Table 1).

### 2.3. Procedure

Data were collected over a 28-day period (from 3 March 2021 to 31 March 2021) through a survey link using the Google Forms platform. The survey was sent to students and faculty through WhatsApp and college e-mail lists. Data analysis was conducted using Stata 16.

### 2.4. Tools and Measures

**Figure 2:**
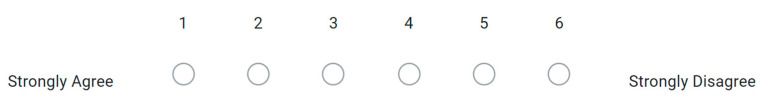
Likert Scale that was implemented for the VAX subscales.

Three sections were included in the Google Forms survey. The first was a self-reported sociodemographic questionnaire. The second was the 12-item Vaccination Attitudes Examination (VAX) Scale [22], which is used to evaluate general attitudes toward vaccinations and is also useful for identifying individuals who are unlikely to vaccinate [22]. It comprises four subscales: “mistrust of vaccine benefits,” “worries about unforeseen future effects,” “concerns about commercial profiteering,” and “preference for natural immunity.” Unlike in previous studies, the participants were asked to focus on the vaccine for COVID-19 rather than all vaccines. Responses were rated on a six-point Likert-type scale ranging from 1 “strongly agree” to 6 “strongly disagree” (Figure 2). The third tool was the standardized Coronavirus Anxiety Scale (CAS) [23], which assesses anxiety associated with the COVID-19 pandemic (Table A1).

**Figure 3:**
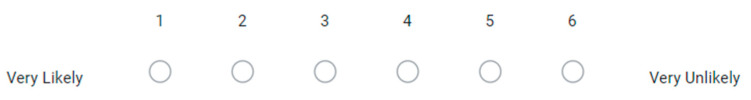
Likert Scale that was implemented for uncertainty and unwillingness to vaccinate against COVID-19.

For each subscale of the VAX tool, the scores were grouped into high (defined as a score of 5–6 on a scale of 1–6), intermediate (score of 3–4), and low (score of 1–2) levels of negative attitudes towards vaccines. We added a further item to assess uncertainty and unwillingness to vaccinate against COVID-19. This item asked “Are you willing to take the vaccine when it is available to you?” Responses ranged from “1–very likely” to “6–very unlikely”(Figure 3). An ordinal variable was coded: (0) intend to vaccinate (responses of 1–2), (1) unsure about whether to vaccinate (responses of 3–4), and (2) unwilling to vaccinate (responses of 5–6). The Cronbach’s alpha value for this scale in our sample was α = 0.84.

Socio-demographic factors included gender (female or male), age group (18–24, 25–34, 35–44, 45–54, 55–64, 65–74, and 75+), nationality (Qatari, North African, Middle Eastern, Gulf Cooperation Council (GCC) country, and South Asian), monthly household income (<5000 QAR, 5000–10,000 QAR, 10,000–15,000 QAR, 15,000–20,000 QAR, 20,000–30,000 QAR, and >30,000 QAR), position (Two groups: faculty members and students. The faculty category was divided into subgroups (lecturer, clinical instructor, assistant professor, associate professor, research assistant, teaching assistant, and other). The student category included undergraduate or postgraduate students), college/department (Medicine, Pharmacy, Health Sciences, Dentistry, Biomedical Research Center), and lastly, the students’ current study year (ranging from Year 1 to Year 6).

Participants were asked yes or no questions about whether they had received a clinical diagnosis of mental illness and whether they had ever been infected with COVID-19, as well as their history of previous vaccine hesitancy. Their history of previous vaccinations was assessed using three questions: (“Have you ever taken a recommended vaccine?”, “Have you ever received the seasonal influenza vaccine in the last three years?”, “Have you received the COVID-19 vaccine?”). 

**Figure 4:**
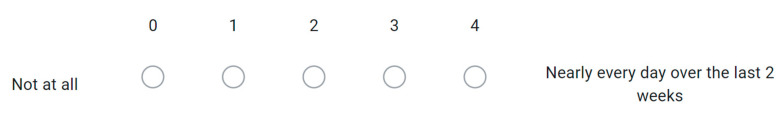
Coronavirus Anxiety Scale (CAS).

The CAS contains five items assessing physical symptoms of anxiety, including but not limited to “dizziness” and “fainting after reading or listening to news about the coronavirus.” The threshold for Generalized Anxiety Disorder was reached by a CAS score of nine or more; thus, participants were classified as either “meeting” (90% sensitivity) or “not meeting” (85% specificity) this threshold [23]. Responses were measured on a 5-point scale ranging from a maximum of “nearly every day” to “not at all” (Figure 4). If a score was 9 or higher, an individual was considered positive for anxiety. Good internal reliability was achieved using the scale (Cronbach’s alpha = 0.88), in addition to good diagnostic viability and construct validity, and it has demonstrated equivalent measurement attempts throughout demographic groups. 

In addition, in order to assess the participants’ confidence in the government and the health service to handle the pandemic, we added the elements “Confidence in government and health system” and “Compliance with government COVID-19 guidelines.” Participants were asked to choose their level of agreement with these statements and to choose one of the following options: never, rarely, sometimes, often, and always. 

### 2.5. Data Analysis

Data analysis was carried using Stata 16.0. Ranges for some sociodemographic variables were collapsed to create larger categories in order to improve detection of sociodemographic effects. This was done for the variables of Age, Nationality, Family Income, Position, and College.

Age was collapsed into 3 groups: 18–34, 35–44, and >45 years old. Nationality was divided into 4 groups: Asians, Europe and North America, Middel East and North Africa (MENA), and sub-Saharan Africa. Family income was split into 4 groups: <10,000 QAR, 10,000–20,000 QAR, 20,000–30,000 QAR, and >30,000 QAR. Position was mainly split into 2 groups: staff and students. The “confidence in the government and health system” answer elements were grouped into low (never and rarely) and high (sometimes, often, and always). Responses to the question on compliance with government COVID-19 guidelines were measured using the same Likert scale mentioned above. We then analyzed this as a binary variable, dividing responses into low (never and rarely) and high (sometimes, often, and always) compliance. The College variable was divided into two categories: College of Medicine and other.

First, we aimed to identify which variables could affect the different subscales on the VAX (Table A1). We implemented the stepwise regression method with the *p*-value set to 0.05. In all equations, the following variables were included: Gender, Age, Nationality, Religion, Mental Status, Family Income, Year, Faculty, College, COVID-19 Status, Compliance with Government COVID-19 Guidelines, Confidence in the Government and Health System, Status of Received Seasonal Flu Vaccine, and Status of Received Recommended Vaccine. The variables selected using the stepwise regression were used as input in the linear regression model; this was followed by a Linktest.

In order to investigate participants’ unwillingness to vaccinate, we implemented a logistic regression model for unwillingness to vaccinate against COVID-19. The means of each subscale of the VAX were used as the input variables. The base outcome was set to “willing to vaccinate.” A Linktest was performed after running the model.

To analyze the responses to the CAS, we implemented a logistic regression model to which the input variables were [Gender, College and Status of Receiving a Recommended Vaccine]. The output variable was binary [Has anxiety/does not have anxiety]. The model was followed by Linktest.

In addition, a Mann–Whitney U-test was performed to compare the means of the CAS scores of the students and faculty.

### 2.6. Ethics

All consent information was displayed on the first page of the survey. Participants had the choice to withdraw at any stage of the survey. This study was approved through Qatar University’s Institutional Review Board (Reference ID: QU-IRB 1494-EA/21) on 1 March 2021.

## 3. Results

### 3.1. Sample Description

Detailed demographic information on our survey sample is presented in Table 2. We received a total of 380 responses, of which 364 were included in our analysis after the inclusion criteria were applied. The majority of participants were students (85.16%), with the highest contribution from Year 2 across the colleges, as they constituted 29.95% of the sample. Most of the respondents (74.12%) were female, and 96.7% of our participants were Muslims, which is expected given the Muslim majority among the population of Qatar at large. About 90% of the participants had never been infected with COVID-19 and only 32.42% of the respondents had been vaccinated at the time of the survey. In addition, almost 28% of the respondents reported having mental health problems, and almost all participants showed high confidence in the government and health system (94.51%) and were compliant with the government’s COVID-19 guidelines (95.6%). Furthermore, 46.43% had received the seasonal flu vaccine.

### 3.2. Attitudes towards Vaccines

**Figure 5:**
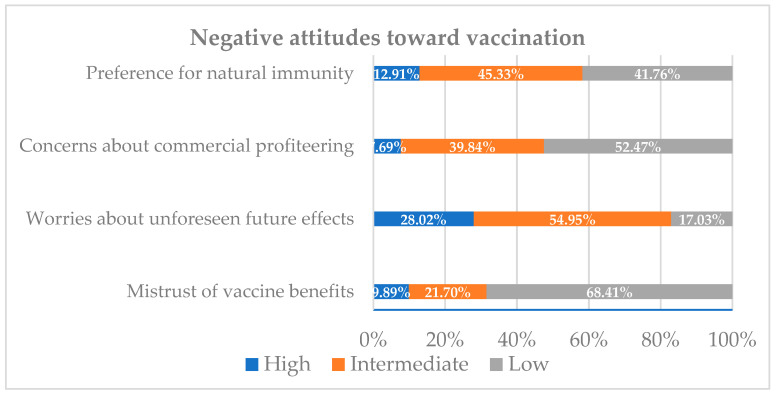
Proportion of the sample reporting high, intermediate, and low negative attitudes towards vaccines.

Of the 364 participants, 9.89% expressed a high mistrust of vaccine safety (i.e., a score of 5–6 on a scale of 1 to 6), while 21.7% were uncertain about their levels of trust (a score of 3–4 out of 6) (Figure 5). Moreover, 28% expressed strong worries about unforeseen side effects, whereas 54.95% expressed moderate worries. Furthermore, 7.69% expressed strong concerns and 39.84% showed moderate concerns about commercial profiteering. Approximately 13% of the participants expressed a strong preference towards natural immunity, whilst 45.33% appeared to believe that natural immunity might be better than a vaccine. Importantly, 68.13% of the participants intended to receive the COVID-19 vaccine once it became available, compared to 17.03% who were uncertain and 14.83% who were unwilling (Figure 5).

#### 3.2.1. Mistrust of Vaccine Benefits (Subscale)

A linear regression model was implemented. The output variable was the average of the three questions that were in the Mistrust of Vaccine Benefit subscale (refer to Table A3). The average score for our participants was 2.6 (refer to Figure 2). On average, men and those who had received the seasonal flu vaccine in the last 3 years scored lower than women and those who had not received the seasonal flu vaccine by almost 0.5 points on the Mistrust of Vaccine Benefits subscale (Coef. = −0.496, CI: −0.883, −0.11) and (Coef. = −0.376, CI: −0.683, −0.068), respectively (Table 3).

Interestingly, we found that as Mistrust of Vaccine Benefits scores decreased with advancement in college years by less than 0.5 points (coef. = −0.152, CI: −0.268, −0.035). Furthermore, individuals from CMED (both students and faculty), on average, scored lower than other health cluster students by almost 0.5 points on the Mistrust of Vaccine Benefits subscale (Coef. = −0.468, CI: −0.793, −0.143) (Table 3).

#### 3.2.2. Worries about Unforeseen Future Effects (Subscale)

A linear regression model was implemented. The output variable was the average of the three questions in the Worries About Unforeseen Future Effects subscale (refer to Table A1). The average score for our participants was 4 (refer to Figure 2). Participants from the College of Medicine, on average, scored 0.5 points lower on this subscale compared to other participants (Coef = −0.307, CI: −0.565 to −0.049) (Table 4). 

#### 3.2.3. Concerns about Commercial Profiteering (Subscale)

A linear regression model was implemented. The output variable was the average of the three questions in the Concerns About Commercial Profiteering subscale (refer to Table A1). The average score for our participants was 2.8 (refer to Figure 2). Participants from the MENA region on average scored higher than participants from other regions by more than 0.5 points (Coef = 0.621, CI: 0.260 to 0.982). Participants from CMED scored on average 0.5 points lower than non-CMED participants on this subscale (Coef = −0.527, CI: −0.784 to −0.270) (Table 5).

#### 3.2.4. Preference for Natural Immunity (Subscale)

A linear regression model was implemented. The output variable was the average of the three questions in the Preference For Natural Immunity subscale (refer to Table A1). The average score for our participants was 3.2 (refer to Figure 2). Participants from CMED and those who received seasonal flu vaccine scored on average 0.5 points lower than participants from other Health Cluster colleges and those who did not receive seasonal flu vaccine on this subscale (Coef. = −0.200, Cl: −0.463, −0.062 and Coef. = −0.434, Cl: −0.698, −0.171, respectively). In addition, individuals who had previously been infected with COVID-19 scored on average 0.5 points higher compared to those who had not been infected (Coef. = 0.614, Cl: 0.222, 1.006) (Table 6).

### 3.3. Unwillingness to Vaccinate against COVID-19

A logistic regression model was implemented. The output variable was binary [Willing to vaccinate/Unwilling to Vaccinate]. The average score for our participants was 2.25 (Fixg4). Participants who scored one point higher on the mistrust of vaccine benefit subscale had a 2.34-fold increase in the odds of being unwilling to vaccinate (OR = 2.34, CI: 1.80 to 3.05). In addition, those who had scored 1 unit higher in the Preference for Natural Immunity subscale had a 1.43-fold increase in unwillingness to vaccinate compared to those who had scored 1 unit less (OR = 1.43, CI: 1.03 to 1.98). The probability of unwillingness to vaccinate against COVID-19 if the participants scored the lowest on all the VAX subscales was 0.16%. Similarly, if the participants had scored the highest, the probability of unwilling to vaccinate against COVID-19 was almost 83% (Table 7).

### 3.4. Corona Virus Anxiety Scale (CAS) against COVID-19

A logistic regression model was implemented. The output variable was binary [Have anxiety/does not have anxiety; based on the CAS score]. Men were 48% less likely to reach the threshold value on the CAS Scale compared to women (OR = 0.519, CI: 0.145 to 1.867). Those in the College of Medicine had 50% lower odds of being positive on the CAS scale compared to those in other Health Cluster colleges (OR = 0.504, CI: 0.207 to 1.231). Furthermore, individuals who had received a recommended vaccine had 57% lower odds of being positive (OR = 0.435, CI: 0.181 to 1.048). However, such findings failed to reject the null hypothesis at this sample (Table 8).

To evaluate the differences in CAS scores between students and faculty in relation to COVID-19, a Mann–Whitney U-test was performed. The mean score of students on the CAS was 2.12, while the mean score for faculty was 2.33. The test revealed that the difference between the students and faculty was not significant (*p* = 0.8861).

## 4. Discussion

This study aimed to examine attitudes and intentions regarding vaccination against COVID-19 among health professions students and faculty in Qatar. The study uncovered various sociodemographic predictors that may affect attitudes toward the COVID-19 vaccine and revealed that certain attitudes shape this population’s hesitancy towards the vaccine. The study reveals that despite the scientific knowledge of the investigated group, several sociodemographic factors interplay with that knowledge and influence attitudes towards vaccination. This is the first study in the Arab region focusing specifically on COVID-19 vaccination attitudes among health professions students and faculty. Focusing on this group is of importance because they have a major contribution in shaping the awareness and knowledge in their communities. Understanding their unique determinants can help in building campaigns tailored to this population’s features, which indirectly influence the general population’s attitudes towards vaccination. 

One of the key findings of this study is that 82.97% of the participants highly or intermediately agreed that taking the vaccine may be accompanied by unforeseen side effects (refer to Figure 5 and Table A4). This suggests that there is a strong concern regarding the vaccine’s safety. Similar findings were reported in Schwarzinger et al.’s study in France, which found that the intention to take the COVID-19 vaccine was mainly influenced by participants’ concerns about safety and side effects [10]. This uncertainty can be explained by the swift process of the COVID-19 vaccine approval compared to previous vaccines. In general, the traditional timeline for vaccine development is 15 to 20 years [24]. However, in order to curb the spread of SARS-CoV-2, the FDA issued emergency approval for certain companies, such as BioNTech, months after their clinical trials.

One of the most frequently circulated myths about the COVID-19 vaccine is that it causes infertility, and many conspiracy theories were built around this idea, including in the Arab region [6]. Such misinformation is likely to contribute to heightened vaccine hesitancy among female participants. Our study found that 11.5% of female participants expressed high mistrust in the vaccine, compared to 5.3% of male participants (refer to Table A2). Contrasting reports of gendered attitudes exist in the literature, with some studies indicating that men are more likely to accept the vaccine [25], while others report higher acceptance among women [15,26]. We assume that differences are the result of factors such as culture, age, education, and socioeconomic status of the investigated group. These factors influence health behaviors in general, and are considered as the main social determinants of health [27].

**Figure 6:**
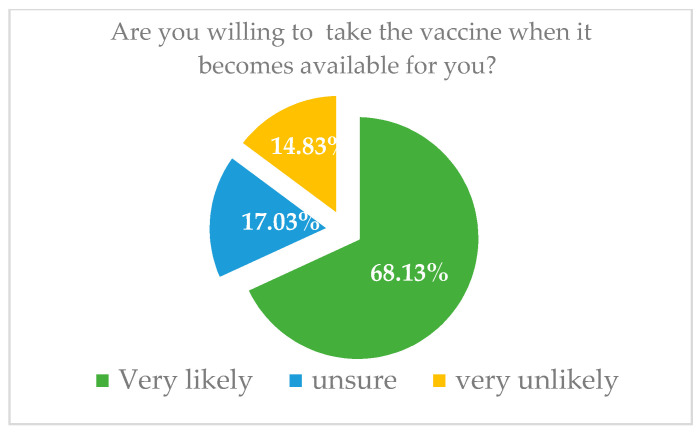
Intent to vaccinate against COVID-19 among all the participants.

The current study indicates that 68.13% of the participants intended to receive the COVID-19 vaccine once it became available, while 17.03% were uncertain and 14.83% were unwilling to take it (refer to Figure 6). These findings differ from the stated intentions of the general population in Qatar, in which the majority (45.2%) expressed uncertainty about taking the COVID-19 vaccine [18]. This difference may be associated with the scientific knowledge of our participants, who are likely to be knowledgeable about the vaccine. Gelle’ et al.’s study in Italy indicates that knowledge contributes to the willingness to take the vaccine [28]. They found that of the total of 3226 undergraduate students from Naples and Rome, 91.9% were keen to receive a COVID-19 vaccination, and more than 80% gave correct answers to questions about the vaccine administration, functioning, and effects on community health. 

This study showed that almost half of the participants (52% of faculty and 40% of students) across the different health professions colleges did not prefer natural immunity (refer to Table A5). This finding aligns with the fact that more than half the participants were likely to receive the COVID-19 vaccine. In addition, 19.5% of individuals who had previously been infected with COVID-19 expressed a high preference for natural immunity (refer to Table A5). This might be explained by participants’ knowledge, their positive experience of the recovery process due to their age category, socioeconomic status, access to health services, and health status in general. In their study on the lived experience of being infected by COVID-19, Shaban et al. found that the contextual factors of the participants’ social and physical environment, together with their individual resources, contributed to their response to being infected and were important mediators of their experiences [29]. 

An important finding in this study is that 76% of the participants who had received the seasonal flu vaccine have scored on average 0.376 less on Mistrust of Vaccine Benefit subscale compared to those who did not receive it (refer to Table A2). This finding can be explained by their positive vaccine experiences and the effectiveness of the flu vaccine. Several studies have reported a positive relationship between having taken the flu vaccine in the past and receiving the COVID-19 vaccine in the present [30,31]. Ayhan et al. found a correlation between hesitancy to take the flu vaccine and hesitancy to take the COVID-19 vaccine [31]. This indicates that the hesitancy is not against the content of the specific vaccine, but rather a reflection of attitudes towards vaccines in general.

Our study also found that those who were enrolled in the College of Medicine had less mistrust of vaccine benefits (refer to Table A2). Similar findings were found by a study conducted with Saudi medical students, which showed a significant linear relationship between vaccination knowledge and attitudes [32]. The influence of medical study and understanding of immunological basics is the main reason. This also aligns with our observation that as students progress throughout the years in the health cluster colleges, there is a decrease in the mistrust of vaccine benefit. 

Interestingly, we found that the probability of being unwilling to vaccinate could leap from 0.16% to almost 91% if all VAX scales progressed from lowest to highest. This indicates how important the subscales are in determining the factors that cause the health-professionals to hesitate and doubt the vaccine. Hence, beliefs and attitudes measured by these four subscales should be targets of policy makers who aim to motivate individuals to increase their willingness to vaccinate. The CDC has approached this in several ways, including encouraging and promoting confidence among healthcare providers in their decision to get vaccinated [33]. UNICEF has also approached the issue of mistrust of vaccine benefits and has recommended that individuals obtain their information regarding the vaccine from trusted resources [34]. This indicates the importance of correct scientific knowledge in shaping attitudes and the intentions to be vaccinated. One example of such relevant knowledge is the recently published finding that the amount of antibodies generated by the SARS-CoV-2 vaccine is higher compared to the amount of antibodies generated by the exposure to the virus itself [35].

To better understand the impact of scientific knowledge and its interplay with other factors shaping vaccine willingness, we recommend future qualitative research that can investigate these factors in more detail and explain how experience in research and in the clinical field may influence attitudes towards vaccination.

Several limitations are present in this study. First, the sample is small due to the low response rate. As numerous studies and surveys were being conducted on COVID-19 at the time, the population was likely suffering from “survey fatigue.” In addition, we acknowledge that our data are not normally distributed and using a linear regression model is not the best option. However, at the time of writing this paper, there have not been any linear regression models that are nonparametric to use as a base to perform such regression model analysis. Furthermore, a low response rate amongst health professions students may be linked to their curricular requirements, making them hesitant to spend time on surveys.

Another limitation is that due to the time constraints, data were collected only from students and faculty at one academic institute in Qatar, which may introduce sampling bias, although Qatar University is both the largest and the national university. Thus, future research should include all health professions faculty and students in all academic institutions in the country to obtain a more generalizable sample. In addition, hesitancy may change over time as people witness the positive impact of the vaccination. Hence, we aim to commence a longitudinal study that evaluates attitudes and hesitancy at different points in time.

## 5. Conclusions

This is the first study to examine attitudes towards and hesitancy regarding the COVID-19 vaccine among health professions faculty and students in the Arab region. The study’s findings suggest that scientific knowledge is a key factor in increasing willingness to take the vaccine. Hence, health professions faculty can play a major role in increasing such knowledge amongst their students, who can, in turn, be major players in distributing evidence-based knowledge in their communities. We believe that health professions students can lead social awareness campaigns and take a key role in controlling infectious disease outbreaks in general, and COVID-19 in particular. In addition, we recommend policy makers make tailored decisions oriented around the VAX subscales in order to increase willingness to vaccinate among the general population. We argue that evidence-based knowledge about the efficiency and safety of the vaccine will help clarify uncertainties and dispel misleading myths in the Arab community, like other communities across the globe. The findings of this research demonstrate the need for more awareness among health care providers of the cultural and social obstacles that may create hesitancy towards COVID-19 vaccination. Such knowledge will enable them to build evidence-based and culturally competent awareness campaigns that make it easier to overcome myths and conspiracy theories.

## Figures and Tables

**Table 1:** Inclusion and Exclusion Criteria.

	Participants	Inclusion Criteria	Exclusion Criteria
1	Students of the QU Health Cluster	Current, enrolled students at one of QU Health Cluster colleges At least 18 years old	Students who are under the age of 18
2	Faculty of the QU Health Cluster BRC staff	Currently employed faculty on research or teaching track at the QU Health Cluster Being employed in one of these ranks: lecturer, assistant professor, associate professor, research assistant, teaching assistant, clinical instructor.	Faculty who do not work in the QU Health Cluster

**Table 2:** Distribution of the sociodemographic data after collapse.

Sociodemographic Variables	Number of Participants	Percentage
Gender		
Female	270	74.18
Male	94	25.82
Age		
18–34	323	88.74
35–44	14	3.85
>=45	27	7.42
Religion		
Islam	352	96.7
Other	12	3.3
Nationality		
Asian	31	8.52
America and Oceania	17	4.67
MENA	310	85.16
Sub-Saharan Africa	6	1.65
Position		
Faculty	54	14.84
Student	310	85.16
Year		
Faculties & Researchers	54	14.84
First Year	56	15.38
Second Year	109	29.95
Third Year	72	19.78
Fourth Year	33	9.07
Fifth Year	28	7.69
Sixth Year	10	2.75
Not Specified	2	0.55
College		
Biomedical Research Center	8	2.2
Dentistry	12	3.3
Health Science	77	21.15
Medicine	184	50.55
Pharmacy	82	22.53
Not Specified	1	0.27
Family Income		
0–10,000 QAR	51	14.01
10,000–20,000 QAR	102	28.02
20,000–30,000 QAR	69	18.96
>30,000 QAR	142	39.01
Covid Status		
Never infected with COVID-19	318	87.36
Has been infected with COVID-19	46	12.64
Mental Illness		
No	336	92.31
Yes	28	7.69
Confidence in the Government and Health System		
High	344	94.51
Low	20	5.49
Compliance with Government COVID-19 Guidelines		
High	348	95.6
Low	16	4.4
Received Seasonal Flu Vaccine		
No	195	53.57
Yes	169	46.43
Received a Recommended Vaccine		
No	64	17.58
Yes	300	82.42
Received a COVID-19 Vaccine		
No	246	67.58
Yes	118	32.42

**Table 3:** Linear regression model of the Mistrust of Vaccine Benefits subscale from the VAX Scale.

Mistrust of Vaccine Benefits	Coef.	95% CI	
Male	−0.496	−0.883	−0.11
Received the seasonal flu vaccine in the last 3 years (‘No’ ref.)	−0.376	−0.683	−0.068
* Year	−0.152	−0.268	−0.035
College (‘Not in College of Medicine’ ref.)	−0.468	−0.793	−0.143
** Constant	3.555	3.183	3.928

* Year 1 to Year 6 students; coded as continuous variable. ** Women who did not receive a seasonal flu vaccine in the last 3 years, are in Year 1, and are not in the College of Medicine scored on average 3.555 on the mistrust of vaccine benefit subscale.

**Table 4:** Linear regression model of Expressed Concerns About Unforeseen Future Effects.

Concerns about Unforeseen Future Effects	Coef.	95% CI	
College (‘Not in College of Medicine’ ref.)	−0.307	−0.565	−0.049
* Constant	4.17	3.986	4.353

* Individuals not from college of Medicine scored on average 4.17 on the worries about unforeseen future effects subscale.

**Table 5:** Linear regression model of Concerns About Commercial Profiteering.

Concerns about Commercial Profiteering	Coef.	95% CI	
College (‘Not in College of Medicine’ ref.)	−0.527	−0.784	−0.270
From MENA Region	0.621	0.260	0.982
* Constant	2.583	2.221	2.945

* Participants not from the MENA region and not in the College of Medicine scored on average 2.583 on concerns about commercial profiteering.

**Table 6:** Linear regression model regarding the Preference for Natural Immunity.

Preference for Natural Immunity	Coef.	95% CI	
College (‘Not in College of Medicine’ ref.)	−0.200	−0.463	−0.062
Had COVID-19	0.614	0.222	1.006
Received Seasonal Flu Vaccine	−0.434	−0.698	−0.171
* Constant	3.403	3.185	3.621

* Participants who were not from College of Medicine, had not been infected with COVID-19, and did not receive a seasonal flu vaccine scored on average 3.403 on the preference for natural immunity subscale.

**Table 7:** Logistic regression model of uncertainty and unwillingness to vaccinate against COVID-19.

Unwillingness to Vaccinate against COVID-19	Odds Ratio	95% CI	
* Mistrust of vaccine benefits	2.34	1.80	3.05
* Worries about unforseen future effects	1.10	0.81	1.50
* Concerns about commercial profiteering	1.32	0.96	1.81
* Preference for natural immunity	1.43	1.03	1.98
Constant	** 0.00	** 0.00	** 0.01

* The subscales (Figure 2) have been rescaled from 0 to 5. ** The actual Constant value is 0.0016 and the CI ranges from 0.0002 to 0.0102. ** The probability of being unwilling to vaccinate if the participant scored the lowest on all these subscales is 0.16%.

**Table 8:** Logistic Regression Model of the Coronavirus Anxiety Scale (CAS).

Coronavirus Anxiety Scale (CAS)	Odds Ratio	95% CI	
Male	0.52	0.15	1.87
College (‘not in College of Medicine’ ref.)	0.50	0.21	1.23
Received Recommended Vaccine in the last Three Years	0.44	0.18	1.05
* Constant	** 0.22	** 0.10	** 0.46

* The actual Constant value is 0.216482 and the 95% CI ranges from 0.102725 to 0.456215. ** The probability of being positive on the CAS scale if the participant was female, was not in the College of Medicine, and had not received a recommended vaccine in the last three years is 17.83%.

## Data Availability

Not applicable.

## Appendix A

**Table A1:** Sociodemographic questionnaire, VAX, and CAS scale implemented in this study.

**The sociodemographic questionnaire includes the following factors:**
Age Gender Nationality [Qatari, North African, Middle-Eastern, GCC country, South Asian, other: ___] Religion [Islam, Christianity, Judaism, Buddhism, Hinduism, other: ___] Position [student undergraduate studies, student graduate studies, professor, clinical instructor, assistant professor, principal investigator, associate professor, research associates, research assistant, teaching assistant] College/department [Medicine, Dentistry, Pharmacy, Human Nutrition, Biomedical Sciences, Public Health, Physical therapy/Rehabilitation, Biomedical Research Center] Current year of study [First Year–Sixth Year] Average family income [<5000 QAR, 5000–10,000 QAR, 10,000–15,000 QAR, 15,000–20,000 QAR, 20,000–30,000 QAR, >30,000 QAR Presence or absence of mental illness Confidence in government and health system Compliance with government COVID-19 guidelines
**Vaccination Attitudes Examination (VAX) Scale consists of four subscales:**
**Mistrust of vaccine benefits** ◦ “I feel safe after being vaccinated” ◦ “I can rely on vaccines to stop serious infectious diseases” ◦ “I feel protected after getting vaccinated” **Worries about unforeseen future effects** ◦ “Although most vaccines appear to be safe, there may be problems that we have not yet discovered” ◦ “Vaccines can cause unforeseen problems in children.” ◦ “I worry about the unknown effects of vaccines in the future.” **Concerns about commercial profiteering** ◦ “Vaccines make a lot of money for pharmaceutical companies, but do not do much for regular people” ◦ “Authorities promote vaccination for financial gain, not for people’s health” ◦ “Vaccination programs are a big con.” **Preference for natural immunity** ◦ “Natural immunity lasts longer than a vaccination” ◦ “Natural exposure to viruses and germs gives the safest protection.” ◦ “Being exposed to diseases naturally is safer for the immune system than being exposed through vaccination.”
**Uncertainty and unwillingness to vaccinate against COVID-19 will be assessed using this item** ◦ Are you willing to take the vaccine when it becomes available for you?
**Coronavirus Anxiety Scale (CAS):**
I felt dizzy, lightheaded, or faint when I read or listened to news about the coronavirus I had trouble falling or staying asleep because I was thinking about the coronavirus I felt paralyzed or frozen when I thought about or was exposed to information about the coronavirus I lost interest in eating when I thought about or was exposed to information about the coronavirus I felt nauseous or had stomach problems when I thought about or was exposed to information about the coronavirus
**History of getting infected with COVID-19 will be assessed using this item:** ◦ “Have you been previously infected with COVID-19”
**History of previous vaccine hesitancy will be measured using this item:** ◦ Have you ever taken a recommended vaccine? ◦ Have you received the seasonal flu vaccine in the last 3 years? ◦ Have you received the COVID-19 vaccine?

**Table A2:** Actual number of participants in Mistrust of Vaccine Benefit subscale.

Mistrust of Vaccine Benefit	High	Intermediate	Low
Gender			
Male	5	15	74
Female	31	64	175
Age			
18–34	35	68	220
35–44	1	4	9
>=45	0	7	20
Religion			
Islam	35	79	238
Other	1	0	11
Nationality			
Asian	0	7	24
Europe, America, and Oceania	1	2	14
MENA	34	69	207
Sub-Saharan Africa	1	1	4
Position			
Faculty	1	13	40
Student	35	66	209
Year			
Faculty and Researchers	1	13	40
First year	8	18	30
Second Year	11	22	76
Third Year	12	14	46
Fourth Year	4	4	25
Fifth Year	0	6	22
Sixth Year	0	0	10
Colleges			
Biomedical Research Center	0	3	5
Dentistry	0	5	7
Health Science	14	17	46
Medicine	14	31	139
Pharmacy	8	23	51
Family Income			
0–10,000 QAR	4	20	27
10,000–20,000 QAR	11	21	70
20,000–30,000 QAR	8	15	46
>30,000 QAR	13	23	106
COVID-19 Status			
Has not been infected with COVID-19	32	66	220
Has been infected with COVID-19	4	13	29
COVID-19 Vaccine Status			
Yes	4	20	94
No	32	59	155
Mental Illness			
No	33	71	232
Yes	3	8	17
Confidence in the Government and Health System			
High	33	72	239
Low	3	7	10
Compliance with Government COVID-19 Guidelines			
High	36	73	239
Low	0	6	10
Received Seasonal Flu Vaccine			
No	26	48	121
Yes	10	31	128
Received a Recommended Vaccine			
No	10	20	34
Yes	26	59	215

Note: total number of participants is 364.

**Table A3:** Actual number of participants in Concerns About Commercial Profiteering subscale.

Concerns about Commercial Profiteering	High	Intermediate	Low
Gender			
Male	5	30	59
Female	23	115	132
Age			
18–34	26	132	165
35–44	0	3	11
>=45	2	10	15
Religion			
Islam	28	145	179
Other	0	0	12
Nationality			
Asian	1	9	21
Europe, America, and Oceania	0	5	12
MENA	27	129	154
Sub-Saharan Africa	0	2	4
Position			
Faculty	2	16	36
Student	26	129	155
Year			
Faculty and Researchers	2	16	36
First year	6	19	31
Second Year	7	47	55
Third Year	7	33	32
Fourth Year	3	14	16
Fifth Year	2	12	14
Sixth Year	0	3	7
Colleges			
Biomedical Research Center	0	4	4
Dentistry	0	5	7
Health Science	11	33	33
Medicine	8	63	113
Pharmacy	9	40	33
Family Income			
0–10,000 QAR	4	29	18
10,000–20,000 QAR	8	43	51
20,000–30,000 QAR	4	27	38
>30,000 QAR	12	46	84
Covid Status			
Has not been infected with COVID-19	23	120	175
Has been infected with COVID-19	5	25	16
COVID-19 Vaccine Status			
Yes	5	35	78
No	23	110	113
Mental Illness			
No	25	138	173
Yes	3	7	18
Confidence in the Government and Health System			
High	25	132	187
Low	3	13	4
Compliance with Government COVID-19 Guidelines			
High	27	137	184
Low	1	8	7
Received Seasonal Flu Vaccine			
No	16	78	101
Yes	12	67	90
Received a Recommended Vaccine			
No	7	32	25
Yes	21	113	166

Note: total number of participants is 364.

**Table A4:** Actual number of participants in Worries About Unforeseen Future Effect subscale.

Worries about Unforeseen Future Effect	High	Intermediate	Low
Gender			
Male	19	50	25
Female	83	150	37
Age			
18–34	94	178	51
35–44	2	7	5
>=45	6	15	6
Religion			
Islam	102	195	55
Other	0	5	7
Nationality			
Asian	5	21	5
Europe, America, and Oceania	3	8	6
MENA	93	167	50
Sub-Saharan Africa	1	4	1
Position			
Faculty	9	31	14
Student	93	169	48
Year			
Faculty and Researchers	9	31	14
First year	17	29	10
Second Year	37	62	10
Third Year	23	38	11
Fourth Year	8	18	7
Fifth Year	6	16	6
Sixth Year	1	5	4
Colleges			
Biomedical Research Center	0	6	2
Dentistry	3	7	2
Health Science	24	44	9
Medicine	46	100	38
Pharmacy	29	42	11
Family Income			
0–10,000 QAR	12	28	11
10,000–20,000 QAR	28	58	16
20,000–30,000 QAR	25	37	7
>30,000 QAR	37	77	28
Covid Status			
Has not been infected with COVID-19	87	178	53
Has been infected with COVID-19	15	22	9
COVID-19 Vaccine Status			
Yes	20	66	32
No	82	134	30
Mental Illness			
No	95	187	54
Yes	7	13	8
Confidence in the Government and Health System			
High	92	191	61
Low	10	9	1
Compliance with Government COVID-19 Guidelines			
High	96	192	60
Low	6	8	2
Received Seasonal Flu Vaccine			
No	61	103	31
Yes	41	97	31
Received a Recommended Vaccine			
No	16	40	8
Yes	86	160	54

Note: Total number of participants is 364.

**Table A5:** Actual number of participants in Preference for Natural Immunity subscale.

Preference for Natural Immunity	High	Intermediate	Low
Gender			
Male	9	41	44
Female	38	124	108
Age			
18–34	26	132	165
35–44	0	3	11
>=45	2	10	15
Religion			
Islam	47	164	141
Other	0	1	11
Nationality			
Asian	4	6	11
Europe, America, and Oceania	2	5	10
MENA	41	141	128
Sub-Saharan Africa	0	3	3
Position			
Faculty	4	22	28
Student	43	143	124
Year			
Faculty and Researchers	4	22	28
First year	6	28	22
Second Year	12	55	42
Third Year	17	30	25
Fourth Year	4	12	17
Fifth Year	0	16	12
Sixth Year	2	2	6
Colleges			
Biomedical Research Center	0	3	5
Dentistry	0	7	5
Health Science	12	34	31
Medicine	23	78	83
Pharmacy	12	43	27
Family Income			
0–10,000 QAR	4	26	21
10,000–20,000 QAR	14	49	39
20,000–30,000 QAR	9	30	30
>30,000 QAR	20	60	62
Covid Status			
Has not been infected with COVID-19	38	144	136
Has been infected with COVID-19	9	21	16
COVID-19 Vaccine Status			
Yes	9	52	57
No	38	113	95
Mental Illness			
No	46	152	138
Yes	1	13	14
Confidence in the Government and Health System			
High	42	157	145
Low	5	8	7
Compliance with Government COVID-19 Guidelines			
High	46	158	144
Low			
Received Seasonal Flu Vaccine			
No	32	94	69
Yes	15	71	83
Received a Recommended Vaccine			
No	7	36	21
Yes	40	129	131

Note: total number of participants is 364.
